# CD163 as a Potential Biomarker of Monocyte Activation in Ischemic Stroke Patients

**DOI:** 10.3390/ijms22136712

**Published:** 2021-06-23

**Authors:** Rosaria Greco, Chiara Demartini, Anna Maria Zanaboni, Elena Tumelero, Alessandra Persico, Elisa Candeloro, Andrea Morotti, Diana Amantea, Cristina Tassorelli

**Affiliations:** 1 IRCCS Mondino Foundation, Via Mondino 2, 27100 Pavia, Italy; chiara.demartini@mondino.it (C.D.); annamaria.zanaboni@mondino.it (A.M.Z.); elena.tumelero@gmail.com (E.T.); alessandra.persico@mondino.it (A.P.); cristina.tassorelli@mondino.it (C.T.); 2 Department of Brain and Behavioral Sciences, University of Pavia, Via Bassi 21, 27100 Pavia, Italy; 3 Department of Neurology and Stroke Unit, Neurology and Stroke Units, ASST-Settelaghi, Ospedale di Circolo Varese, Viale Borri 57, 21100 Varese, Italy; elisa.candeloro@asst-settelaghi.it; 4 Neurology Unit, Department of Neurological Sciences and Vision, ASST-Ospedali Civili, 25121 Brescia, Italy; andrea.morotti85@gmail.com; 5 Section of Preclinical and Translational Pharmacology, Department of Pharmacy, Health and Nutritional Sciences, University of Calabria, 87036 Rende, Italy; diana.amantea@unical.it

**Keywords:** acute ischemic stroke, CD163+, CD80+, cytokines, peripheral blood monocytes

## Abstract

In ischemic stroke patients, a higher monocyte count is associated with disease severity and worse prognosis. The complex correlation between subset phenotypes and functions underscores the importance of clarifying the role of monocyte subpopulations. We examined the subtype-specific distribution of the CD163+ and CD80+ circulating monocytes and evaluated their association with the inflammatory status in 26 ischemic stroke patients and 16 healthy controls. An increased percentage of CD163+/CD16+ and CD163+/CD14++ events occurred 24 and 48 h after a stroke compared to the controls. CD163+ expression was more pronounced in CD16+ non-classical and intermediate monocytes, as compared to CD14+ classical subtype, 24 h after stroke. Conversely, the percentage of CD80+/CD16+ events was unaffected in patients; meanwhile, the percentage of CD80+/CD14+ events significantly increased only 24 h after stroke. Interleukin (IL)-1beta, TNF-alpha, and IL-4 mRNA levels were higher, while IL-10 mRNA levels were reduced in total monocytes from patients versus controls, at either 24 h or 48 h after stroke. The percentage of CD163+/CD16+ events 24 h after stroke was positively associated with NIHSS score and mRS at admission, suggesting that stroke severity and disability are relevant triggers for CD163+ expression in circulating CD16+ monocytes.

## 1. Introduction

The pathobiology of an ischemic stroke is influenced by the activation of immune responses occurring both locally in the brain and in the peripheral circulation. Accordingly, evidence from animal models and patients highlights that ischemic insult results in rapid activation of local microglia, followed by stimulation and cerebral recruitment of circulating monocytes that differentiate into macrophages or dendritic cells, influencing the progression of ischemic damage [1,2,3,4]. Thus, monocytes modulate the immune response by polarizing toward diverse phenotypes that display tangled, often paradoxical, and still poorly characterized functions. Human monocytes can be classified according to their relative expression of the surface molecules CD14 (lipopolysaccharide receptor complex component) and CD16 (FCγRIII immunoglobulin receptor): 80–90% of circulating monocytes, referred to as “classical” monocytes, express high levels of CD14 but no CD16; meanwhile, the population expressing CD16 includes “intermediate” monocytes (expressing high levels of CD14) and “nonclassical” monocytes characterized by low CD14 expression [5,6,7]. Although recent work has delineated diverse phenotypes, their specific inflammatory role has not been clarified, whereby redundant functions or contradictory findings have been reported in different inflammatory conditions [8,9,10]. Several studies suggest that the disease stage and some risk factors can differentially affect specific monocyte subsets; therefore, these cells may be a biomarker predictive of outcomes. In the context of ischemic stroke, a higher monocyte count is associated with disease severity and adverse prognosis [11,12,13]. The few studies performed to date have revealed that the more abundant (CD14+) classical monocyte subset triggers detrimental effects; on the contrary, the less-represented (CD16+) populations may exert beneficial functions [14,15]. This is consistent with the concept that classical monocytes promote damage by producing inflammatory cytokines (i.e., TNF-alpha, IL-1beta, and IL-6) [16], with evidence that their elevation in the blood of acute stroke patients is independently associated with poor outcomes [17,18]. Conversely, CD16+ intermediate and nonclassical monocytes have been reported to be inversely related to poor functional and histological outcomes and to mortality, respectively [18]. In a recent clinical study, we reported a significant elevation of the percentage of CD14++/CD16+ intermediate and CD14+/CD16+ non-classical monocyte subsets, as well as their relative expression of the cannabinoid receptor-2 (CB2), 24 and 48 h after stroke. By contrast, the percentage of CD14++/CD16– events (corresponding to classical monocyte subtype) was not affected [19]. Interestingly, we also found that the increase in CB2 protein expression in CD16+ monocytes positively correlated with stroke severity, likely representing a compensatory response to limit damage if considering their anti-inflammatory and pro-angiogenic functions [20,21]. 

Elevated CD14++/CD16− monocytes predict cardiovascular events, whereas the percentage of monocytes expressing CD16 is negatively associated with carotid artery intima-media thickness at baseline [22]. However, there is an overlap between these specific subsets when they are defined just by CD14+/CD16+ expression; thus, a clear discrimination and, accordingly, characterization of these subsets is challenging. Moreover, different monocyte subsets seem to have distinct biological functions depending on the clinical or inflammatory context. For instance, CD14++/CD16− monocytes might cause inflammation that damages the fibrous cap of atherosclerotic plaques and might thus be associated with an increased risk of clinical events; CD16-expressing monocytes might play a role in determining the plaque size, possibly even having a protective or reparative rather than plaque-promoting function [22]. Recent studies have shown a substantial heterogeneity of immune infiltrates, raising a key question regarding the functional role of each cellular phenotype recruited to the ischemic brain.

Several biomarkers are associated with the M1/M2 profiles of human monocytes [23,24]. CD80 (B7-1), a costimulatory signal for T cell activation and survival, is mainly expressed on M1 macrophages. By contrast, CD163, the high-affinity scavenger receptor for the hemoglobin–haptoglobin complex, is selectively expressed on M2 monocytes/macrophages, where it participates in clearance of hemoglobin/haptoglobin complexes and tissue protection from oxidative damage [25]. Many anti-inflammatory signals upregulate CD163, while pro-inflammatory signals downregulate its expression [24,26]. Starting from 5–6 days after a stroke in humans, CD163+ cells accumulate in the ischemic brain parenchyma, where they may also paradoxically acquire a pro-inflammatory phenotype [27], as also noted in other pathological contexts [28,29,30]. This highlights the complexity of the correlation between subset phenotypes and functions, underscoring the importance of clarifying the role of specific monocyte subpopulations, beyond the surface expression of CD14 and CD16. In fact, immunomodulatory approaches aimed at targeting specific cell populations represent potential effective treatments for stroke and would significantly benefit from a better understanding of the molecular events underlying stroke-induced inflammation [31]. The occurrence of distinct monocyte subsets has not been fully investigated in ischemic stroke patients, even though monocytes (as well as macrophages) have been recognized as important players of the inflammatory response. Herein, using flow cytometry, we examined the subtype-specific distribution of CD163+ and CD80+ circulating monocytes in ischemic patients during the acute phase and evaluated the global inflammatory status by analyzing cytokine gene expression in total monocytes.

## 2. Results

### 2.1. CD163 and CD80 Expression in Peripheral Blood Monocytes

**Figure 1:**
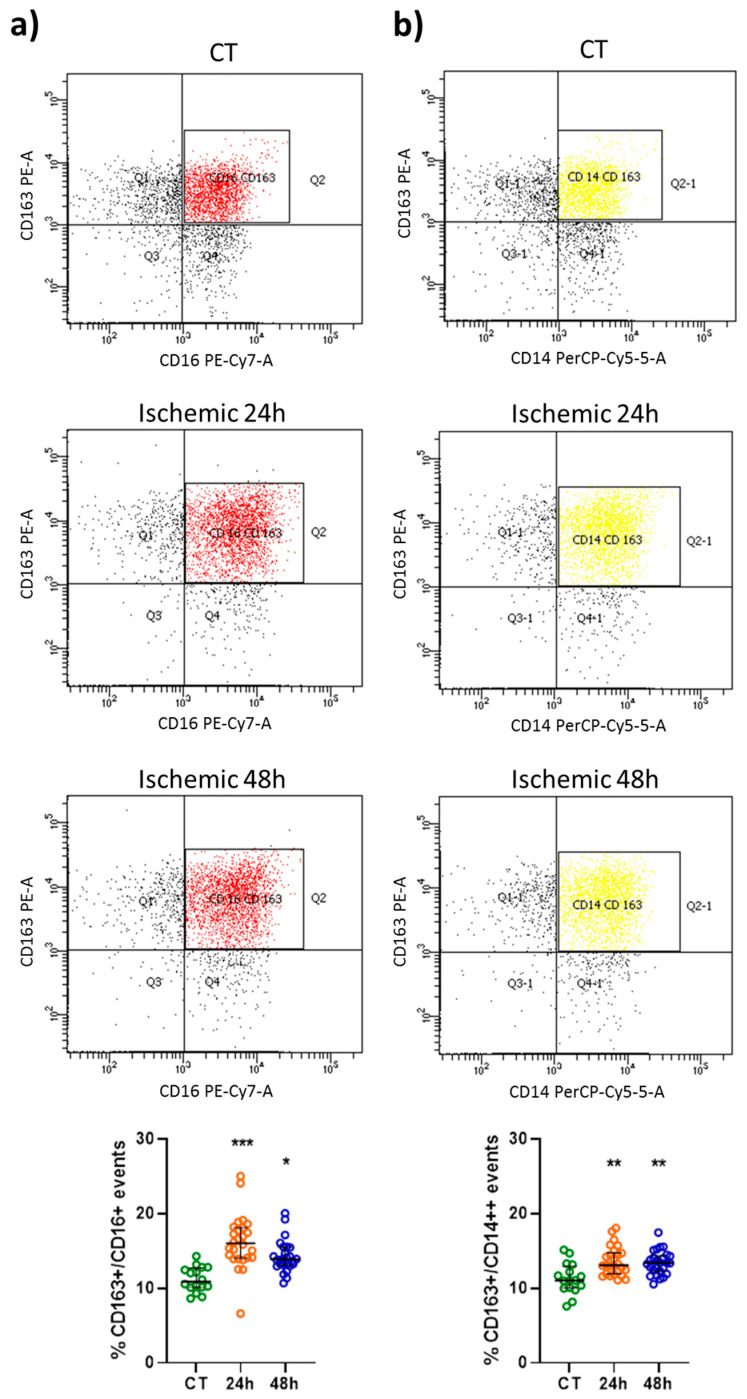
Representative flow cytometry density plots and scatter plots showing the percentage of CD163+/CD16+ (red) (**a**) and CD163+/CD14++ (yellow) (**b**) events in healthy subjects (CT; *n* = 16) and in patients 24 and 48 h after an ischemic stroke (*n* = 26). Data are shown as the median and interquartile range. A Kruskal–Wallis test, followed by Dunn’s post-hoc test: * *p* < 0.05, ** *p* < 0.01, and *** *p* < 0.001 vs. CT.

**Figure 2:**
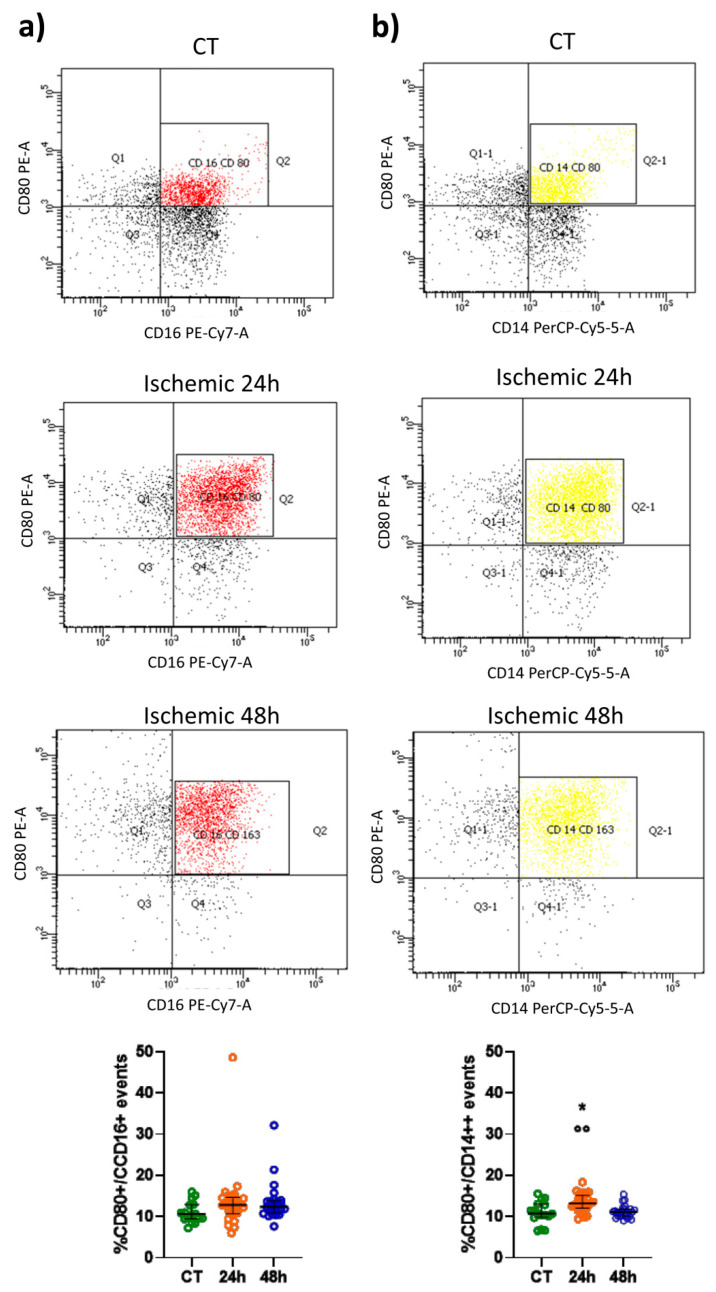
Representative flow cytometry density plots and scatter plots showing the percentage of CD80+/CD16+ (red) (**a**) and CD80+/CD14++ (yellow) (**b**) events in healthy subjects (CT; *n* = 16) and in patients 24 and 48 h after an ischemic stroke (*n* = 26). Data are shown as the median and interquartile range. A Kruskal–Wallis test, followed by Dunn’s post-hoc test: * *p* < 0.05 vs. CT; Wilcoxon’s signed-rank test: °° *p* < 0.01 vs. 48 h.

An increased percentage of CD163+/CD16+ and CD163+/CD14++ events was found 24 and 48 h after an ischemic stroke when compared to control subjects (CT) (Figure 1a,b). The percentage of CD16+ cells expressing CD163 (median = 16.01%, IQR = 14.05–18.12) was higher (*p* < 0.01) than the percentage of CD14++ cells expressing this surface molecule (median = 12.46%, IQR = 11.88–14.63) 24 h after stroke, but not at a later time-point (i.e., 48 h after the insult). Thus, elevation of CD163 expression is more pronounced in CD16+ non-classical and intermediate monocytes at least in the acute phase. Conversely, the percentage of CD80+/CD16+ events was unaffected after stroke (Figure 2a), whereas the percentage of CD80+/CD14++ events was significantly increased 24 h after the insult as compared to CT (Figure 2b).

### 2.2. Cytokines Gene Expression 

**Figure 3:**
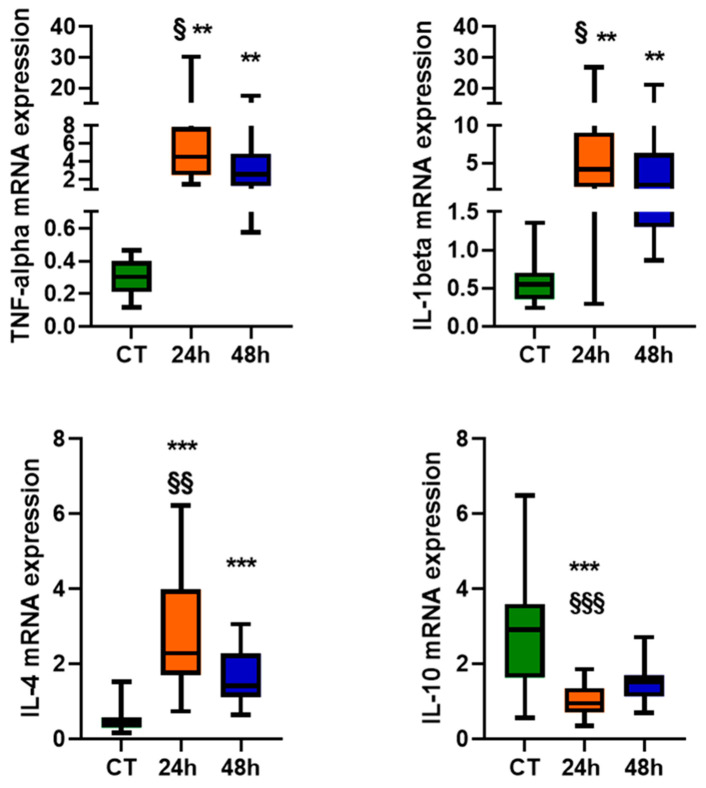
Cytokines’ mRNA levels, expressed as relative quantification (RQ) in the monocytes of healthy subjects (CT; *n* = 16) and patients 24 and 48 h after an ischemic stroke (*n* = 24–26). Data are shown as the median and the minimum and maximum values. A Kruskal–Wallis test, followed by Dunn’s post-hoc test: ** *p* < 0.01 and *** *p* < 0.001 vs. CT; Wilcoxon’s signed-rank test: § *p* < 0.05, §§ *p* < 0.01, and §§§ *p* < 0.001 vs. 48 h.

Increased IL-1beta, TNF-alpha, and IL-4 mRNA levels were observed in the total monocytes from ischemic stroke patients at either 24 or 48 h after the insult, compared to CT, with no significant difference between the two time points (Figure 3). By contrast, a significant decrease in IL-10 gene expression was found 24 and 48 h after an ischemic stroke compared to CT subjects.

### 2.3. Correlation between the Percentage CD163+/CD16+ Events and Stroke Severity

**Figure 4:**
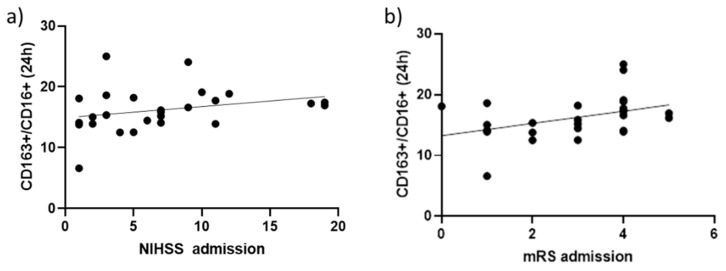
Correlation of the percentage of CD163+ events in CD16+ monocytes with NIHSS (**a**) and mRS (**b**) score at admission: *r*  =  0.39, *p*  =  0.05 and *r*  =  0.39, *p*  =  0.047 (Spearman’s rank correlation coefficient), respectively.

The percentage of CD163+/CD16+ events 24 h after an ischemic stroke was positively associated with the National Institute of Health Stroke Scale (NIHSS) score (Figure 4a; *r*  =  0.39, *p*  =  0.05) and the modified Rankin Scale (mRS) at admission (Figure 4b; *r*  =  0.39, *p*  =  0.047), suggesting that stroke severity and disability are relevant triggers for the expression of CD163+ on circulating CD16+ monocytes. No significant correlation was reported between CD80+ subsets and stroke severity/disability (data not shown).

## 3. Discussion

Following a stroke, a considerable stimulation of the immune system occurs, resulting in beneficial or detrimental effects on ischemic outcomes. Immunity and inflammation play pivotal roles in the pathogenesis of acute stroke, and the immune system is considered a promising target to limit brain damage progression during or after stroke [32,33]. Previous studies have suggested that several clinical implications may be related to the modulation of different surface receptors in monocytes or their capacity to present antigens or produce inflammatory cytokines [18]. Additionally, Kaito et al. [17] reported that distinct monocyte subsets may be associated with medical complications during the acute and subacute phases of an ischemic stroke. For instance, intermediate monocytes may be involved in brain tissue damage in progressing infarction, while nonclassical monocytes seem to be associated with infectious complications after stroke. Furthermore, another study suggested that CD16+ nonclassical and intermediate monocytes are inversely correlated with mortality and poor functional and histological outcomes, respectively [18]. Therefore, the development of “immunomodulatory strategies” that specifically modulate the function of selected monocyte subsets after ischemia may constitute a promising therapeutic strategy.

CD163 has been reported to predominantly exert anti-inflammatory functions [34], being selectively expressed on M2 macrophages and monocytes [35]. CD163 expression is upregulated in several inflammatory diseases, although knowledge of the pathological role of this receptor is incomplete. The immune effects of CD163 are rather complex and are not limited to downregulating inflammation. CD163 stimulation, indeed, has also been reported to induce inflammatory responses in rodent and human macrophages [29,36]. By contrast, CD80 (B7-1), a costimulatory signal for T cell activation and survival, is preferentially expressed on inflammatory M1 monocytes/macrophages. 

The present study was conducted to delineate the CD163 and CD80 profiles in the circulating monocytes of patients after an ischemic stroke and healthy individuals to provide information about their phenotypic distribution and putative function. Additionally, since the gene expression of specific cytokines is modulated after an ischemic stroke in peripheral blood cells, we also assayed pro- and anti-inflammatory cytokine mRNA levels in circulating monocytes. Thus, we detected a significant increase in the percentage of CD163+/CD16+ and CD163+/CD14++ events 24 and 48 h after stroke, compared to healthy individuals. Due to the different subtypes of ischemic stroke and the high variability in lesion volume (detected by CT or NMR imaging), together with the small sample size, we were unable to establish a correlation between CD163+/CD16+ events and these parameters. However, only the percentage of CD163+/CD16+ events counted 24 h after an ischemic stroke was positively associated with NIHSS score and mRS at admission. Considering the beneficial role of CD163+/CD16+ monocytes, our evidence suggests that stroke severity and disability may represent relevant triggers for the expression of CD163+ on circulating CD16+ subsets. Although CD163 is considered an M2 marker, it seems likely that only a subpopulation of CD163+ is M2; whereby, a distinct CD163+ subpopulation may be defined [37]. CD163-expressing macrophages are also involved in the resolution of inflammation by secreting anti-inflammatory cytokines in response to inflammatory events [38]. This is consistent with our evidence of a significant increase in the percentage of CD163+/CD16+ events 24 h after stroke compared to the CT group, while no significant difference in the percentage of CD80+/CD16+ events occurred. 

An upregulation of TNF-alpha, IL-1beta, and IL-4 gene expression was found in total monocytes 24 and 48 h after an ischemic stroke compared to healthy conditions. Meanwhile, a significant reduction in IL-10 gene expression was found only 24 h after the insult. This is consistent with the evidence that inflammatory cytokine expression is modulated by a stroke in both rodent and human leukocytes [33,39,40,41,42]. Cytokine mRNA levels were measured in total monocytes, which did not allow to discriminate the contribution of each cellular phenotype or of other immune cells [43]. Moreover, cytokine extracellular (plasma/serum) levels may strongly depend on the time point after the insult [44,45] and may be affected by stroke subtype [46], though the latter point would require a larger sample size to be validated. In fact, cytokines are known to stimulate CD163 shedding [34], while inflammatory stimuli, including Lipopolysaccharide and IL-4, downregulate CD163 expression in human monocytes [47]. In stroke patients, peripheral blood ADAM17 activity and soluble CD163 levels are elevated, whereby CD163 can be shed from the plasma membrane via the metalloprotease ADAM17 to generate a soluble peptide with lympho-inhibitory properties [43]. In fact, elevated levels of soluble CD163 in the peripheral blood of ischemic stroke patients are negatively associated with lymphocyte counts [43], and these levels may be involved in the resolution of inflammation by mechanisms that are not yet fully understood [48].

Our findings show an increased expression of CD163+ on CD16+ and CD14+ monocytes that was significantly more pronounced on CD16+ subsets at 24 h. Accordingly, elevated mRNA levels of CD163, probably in response to stroke-induced increases in circulating free hemoglobin, were found in the peripheral cells of ischemic patients [49]. 

Recently, it was reported that de novo expression of CD163 by activated microglia/macrophages and CD163+-infiltrating monocytes occurs in both hemorrhagic and non-hemorrhagic brain lesions in patients, highlighting the possibility of targeting CD163-dependent functions for the treatment of both types of lesion [50]. CD163 is almost exclusively expressed on monocytes and perivascular macrophages and may participate in the modulation of inflammatory responses and in angiogenetic functions [29]. Additionally, a functional analysis of the genes overexpressed in CD163+ CNS border-associated macrophages after brain ischemia has been reported in rats, supporting the activation of neovascularization processes and of the hypoxia-inducible factor-1 pathway in the acute phase [51]. 

Furthermore, high CD163 expression in macrophages is typically observed in tissues responding to inflammation, thus the nonshed membrane form of CD163 represents a target for drugs to be directed at macrophages under inflammatory conditions. By contrast, the plasma level of soluble CD163 is increased in a large spectrum of acute and chronic inflammatory disorders and has been suggested to be a reliable inflammatory biomarker [34].

## 4. Materials and Methods

### 4.1. Patients and Sample Collection

This study is a cross-sectional case control study conducted on 25 patients of both sexes with a diagnosis of acute ischemic stroke (within the first 48 h from symptom onset) and 16 age-matched healthy CT. Patients were recruited from the U.C. Malattie Cerebrovascolari e Stroke Unit, IRCCS Fondazione Mondino, from October 2017 to September 2020. The participants’ age ranged from 46 to 95 years. Written informed consent was obtained from both patients and control subjects. The patients who presented with hemorrhagic stroke, systemic inflammatory diseases, or those treated with immunosuppressant drugs were excluded from the study. All CT underwent complete physical and instrumental examination, as well as inflammation biomarkers to rule out the presence of any co-existing pathology. None of the CT had a previous history of inflammation or reported chronic use of any medication. Moreover, the CT were advised to avoid the occasional intake of anti-inflammatory drugs during the seven days preceding the blood sample collection.

The study was approved by the local ethics committee (N. p-20170026158) and was conducted following the principles of the Declaration of Helsinki. All patients were assessed for stroke severity and degree of disability using NIHSS and mRS, respectively. The diagnosis of stroke was always made by the same experienced neurologist and confirmed using computed tomography or magnetic resonance imaging.

Peripheral blood was obtained by venipuncture and processed immediately as described below. Analyses in monocytes were performed by flow cytometry (for surface marker characterization) and by rtPCR (for cytokine gene expression), 24 and 48 h after the ischemic stroke.

The subjects’ characteristics are summarized in Table 1.

### 4.2. Isolation of Peripheral Blood Mononuclear Cells (PBMCs) 

Twenty milliliters of blood was taken from a peripheral vein at 24 h (24 ± 2 h) and 48 h (48 ± 2 h) after hospital admission for ischemic stroke. The blood from participants was collected in ethylenediamine tetra-acetic acid (EDTA)-containing tubes. Blood samples were diluted at a 1:1 ratio with phosphate-buffered saline (PBS) 1X (Sigma). Diluted blood samples were slowly loaded onto 15 mL of Ficoll separating solution and centrifuged at 800× *g* without break for 30 min at room temperature. PBMCs were collected from the middle white monolayer, washed twice in sterile PBS 1X, and centrifuged at 300× *g* for 15 min.

### 4.3. Monocyte Isolation

Untouched monocytes were isolated from PBMCs, collected as described above, using the Pan Monocyte Isolation Kit and a MidiMACS™ Separator (MiltenyiBiotec). To this end, PBMCs were resuspended in PBS containing 0.5% bovine serum albumin and 2 mM EDTA. Non-monocytes, such as T cells, NK cells, B cells, dendritic cells, and basophils, were indirectly magnetically labeled using a cocktail of biotin-conjugated antibodies along with aFcR blocking reagent and Anti-Biotin MicroBeads. Depletion of the magnetically labeled cells allowed the isolation of high-purity unlabeled monocytes.

### 4.4. Flow Cytometry Analysis

Monocytes were centrifuged at 300× *g* for 15 min and then resuspended in sterile PBS and counted. After 10 min of preincubation with human FcR Blocking Reagent (MiltenyiBiotec) to block non-specific binding to the Fc receptor, a total of 1 × 10^5^ monocytes were incubated for 30 min at 4 °C in the dark with the following antibodies: Peridinin Chlorophyll Protein Complex (PerCP)-conjugated anti-human CD14 (BD Biosciences, 1:50), R-phyco-erythrin-cyanine7 (PE-Cy7)-conjugated anti-human CD16 (BD Biosciences, 1:50), and R-phycoerythrin (PE)-conjugated CD163 (BD Biosciences, 1:50) or, alternatively, R-phycoerythrin (PE)-conjugated CD80 (BD Biosciences, 1:50). Unstained cells, i.e., incubated in the absence of fluorescently labeled antibodies, were used to assess background fluorescence to set voltages and negative gates appropriately. Single stained samples were also prepared to evaluate fluorescence spread, to compensate for spectral overlap and to set positive gating. 

Fluorescent cellular events were assessed by flow cytometry using a BD FACS Canto flow cytometer (BD Biosciences). For each sample, 1 × 10^4^ events were acquired and monocytes were assumed to be CD14+ and/or CD16+; meanwhile, CD163 or CD80 expression was assessed in classical (CD14++/CD16–) and non-classical/intermediate (CD14+/CD16+) subsets. Data are reported as percentage of total acquired events.

### 4.5. Gene Expression (rtPCR) in Monocytes

Total RNA from isolated monocytes was obtained using a standard procedure (Zymo Research) and RNA quality was assessed using a spectrophotometer (Nanodrop™ Thermo Fisher Scientific); cDNA was generated using an iScript cDNA Synthesis Kit (Bio-Rad) following the supplier’s instructions. The gene expression of pro-inflammatory and anti-inflammatory cytokines was analyzed using the Fast Eva Green Supermix (Bio-Rad). The primer sequences obtained from the AutoPrime software (http://www.autoprime.de/AutoPrimeWeb; accessed on 4 December 2017) are reported in Table 2. Ubiquitin C (UBC), whose expression remained constant in all experimental groups, was used as housekeeping gene. The amplification was performed with a light Cycler 480 Instrument rt-PCR Detection System (Roche) following the supplier’s instructions. All samples were assayed in triplicate and gene expression levels were calculated according to the 2^−∆∆Ct^ = 2^−(Ct gene − Ct housekeeping gene)^ formula by using Ct values.

### 4.6. Statistical Analysis

All data were analyzed using Prism 8 version 23.0 (GraphPad Software). The differences in demographic variables between the CT and patients were evaluated with a Mann–Whitney test. Differences between categorical variables were analyzed using the Chi-square test. The nonparametric Kruskal–Wallis test, followed by Dunn’s multiple comparison test, was used to compare cytokine gene expression and monocyte flow cytometry analysis in patients and healthy individuals. Spearman’s rank correlation coefficient was used to assess the correlation between NIHSS score or mRS with CD163+/CD16+ or CD163+/CD16+ with IL-4 gene expression. The reported probability values are two-tailed, and *p* < 0.05 was considered statistically significant. Data are presented as the median with the interquartile range (IQR) or the minimum and maximum values for non-normally distributed data.

## 5. Conclusions

Given the importance of monocytes in the pathobiology of an ischemic stroke, delineating the functional and phenotypic characterization of their subsets is a critical requirement for both prognostic and therapeutic aims. Our findings suggest that CD163 may be a potential biomarker of monocyte/macrophage activation associated with the severity of the disease, highlighting its putative role as a target for immunomodulatory treatment strategies. Nevertheless, the relevance of our findings should be confirmed on a larger sample size and at different time points after the insult.

## Figures and Tables

**Table 1:** Demographic and clinical variables.

	Healthy Controls	Patients	*p*-Value
Subjects (*n*)	16	26	
Age (median with range)	83 (94–46)	77 (95–52)	0.98 ^a^
Gender (F/M)	12/16	16/26	0.07 ^b^
Hypertension (%)	2/16 (12.5%)	14/26 (53%)	
Dyslipidemia (%)	0	5/26 (19.2%)	
Diabetes mellitus (%)	0	6/26 (23%)	
Obesity (%)	0	6/26 (23%)	
Smoking (%)	0	6/26 (23%)	
Alcohol abuse (%)	0	1/26 (3.8%)	
Drug abuse (%)	0	1/26 (3.8%)	
Prior stroke (%)	0	3/26 (11.5%)	
Thrombolysis (%)	0	11/26 (42%)	
Thrombectomy (%)	0	10/26 (38.5%)	
Thrombolysis and Thrombectomy (*%*)	0	5/26 (19.2%)	
NIHSS score (entrance) (median with range)	0	5.5 (1–19)	
NIHSS score (discharge) (median with range)	0	1.5 (1–4)	
mRS entrance (median with range)	0	3 (0–5)	
mRS discharge (median with range)	0	2 (0–5)	
TOAST			
Stroke of undetermined cause	0	6/26 (26%)	
Large artery atherosclerosis	0	5/26 (19.2%)	
Lacunar	0	1/26 (3.8%)	
Stroke of other determined cause	0	7/26 (26.69%)	
Cardioembolism	0	7/26 (26.9%)	

mRS: modified Rankin Scale; NIHSS: National Institute of Health Stroke Scale; TOAST: Trial of Org 10,172 in Acute Stroke Treatment. ^a^ Mann–Whitney test; ^b^ Chi-square test.

**Table 2:** Primer sequences.

Gene	Forward Primer	Reverse Primer
UBC	AGAGGCTGATCTTTGCTGGA	GTGGACTCTTTCTGGATG
IL-1beta	CCTGAGCTCGCCAGTGAAAT	TCGTGCACATAAGCCTCGTT
TNF-alpha	CACAGTGAAGTGCTGGCAAC	ACATTGGGTCCCCCAGGATA
IL-4	CGTCTTTAGCCTTTCCAAGAA	CGAGTTGACCGTAACAGA
IL-10	GTCATCGATTTCTTCCCTGTG	ACTCATGGCTTTGTAGATGCCT

## Data Availability

The data presented in this study are available from the ZENODO repository (10.5281/zenodo.4916312).
